# Supplementary material for: Environmental enrichment enhances patterning and remodeling of synaptic nanoarchitecture as revealed by STED nanoscopy
Source: eLife. 2022 Feb 23;11:e73603. doi: 10.7554/eLife.73603 (PMC8903838; doi:10.7554/eLife.73603)

Figure 5–source data 1: Images of all analyzed perforated PSD95 assemblies

Sections of STED time-lapse images of spines (magenta) and PSD95 assemblies (green) utilized for the analysis of the PSD95 nanopattern. (A) EE housed mice and (B) Ctr housed mice. Maximum intensity projection (MIP). Scale bar: 500 µm.

A1 - EE

0 min **30 min** **60 min**

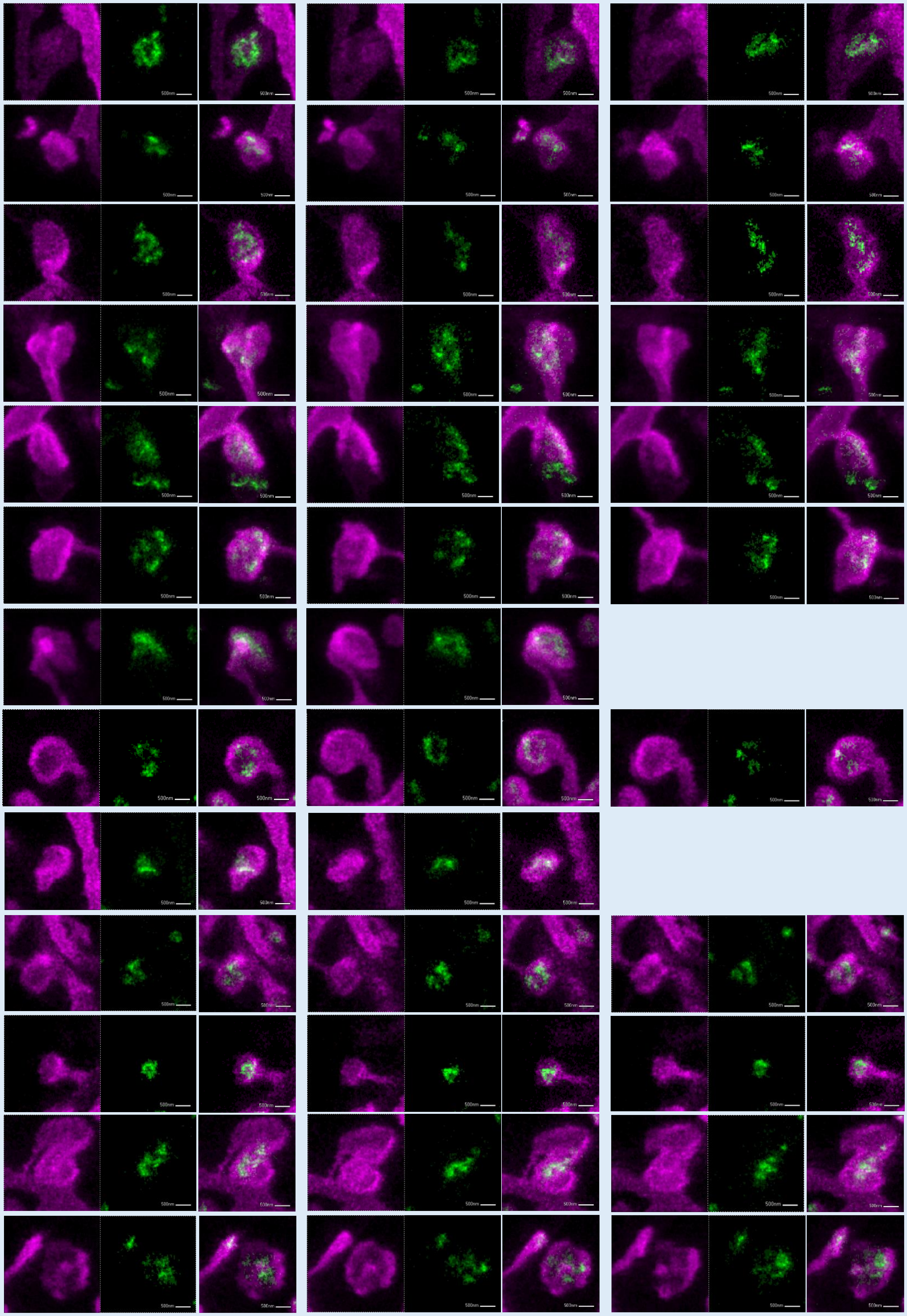

A2 - EE

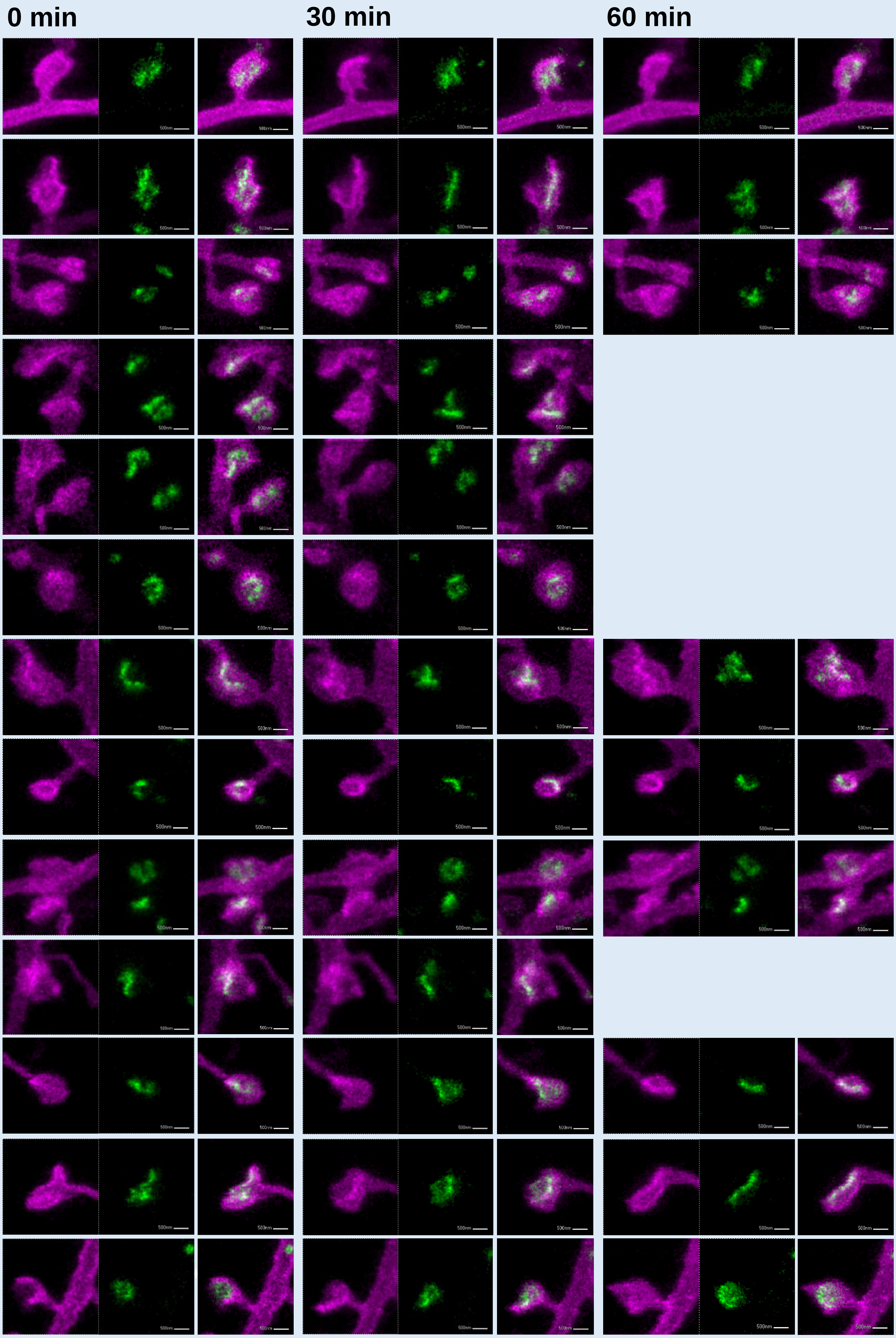

A3 - EE

0 min **30 min** **60 min**

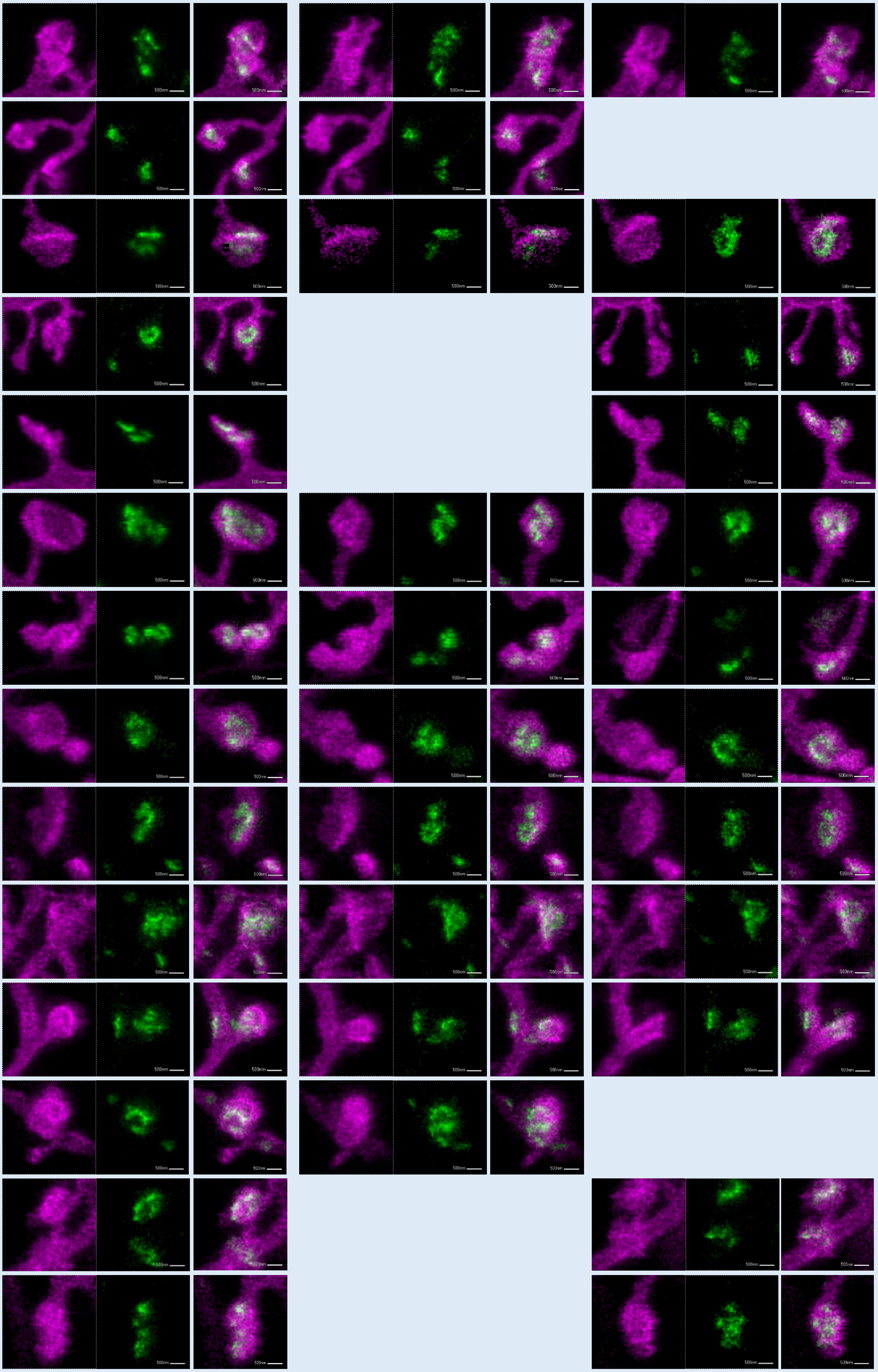

A4 - EE

0 min **60 min** **120 min**

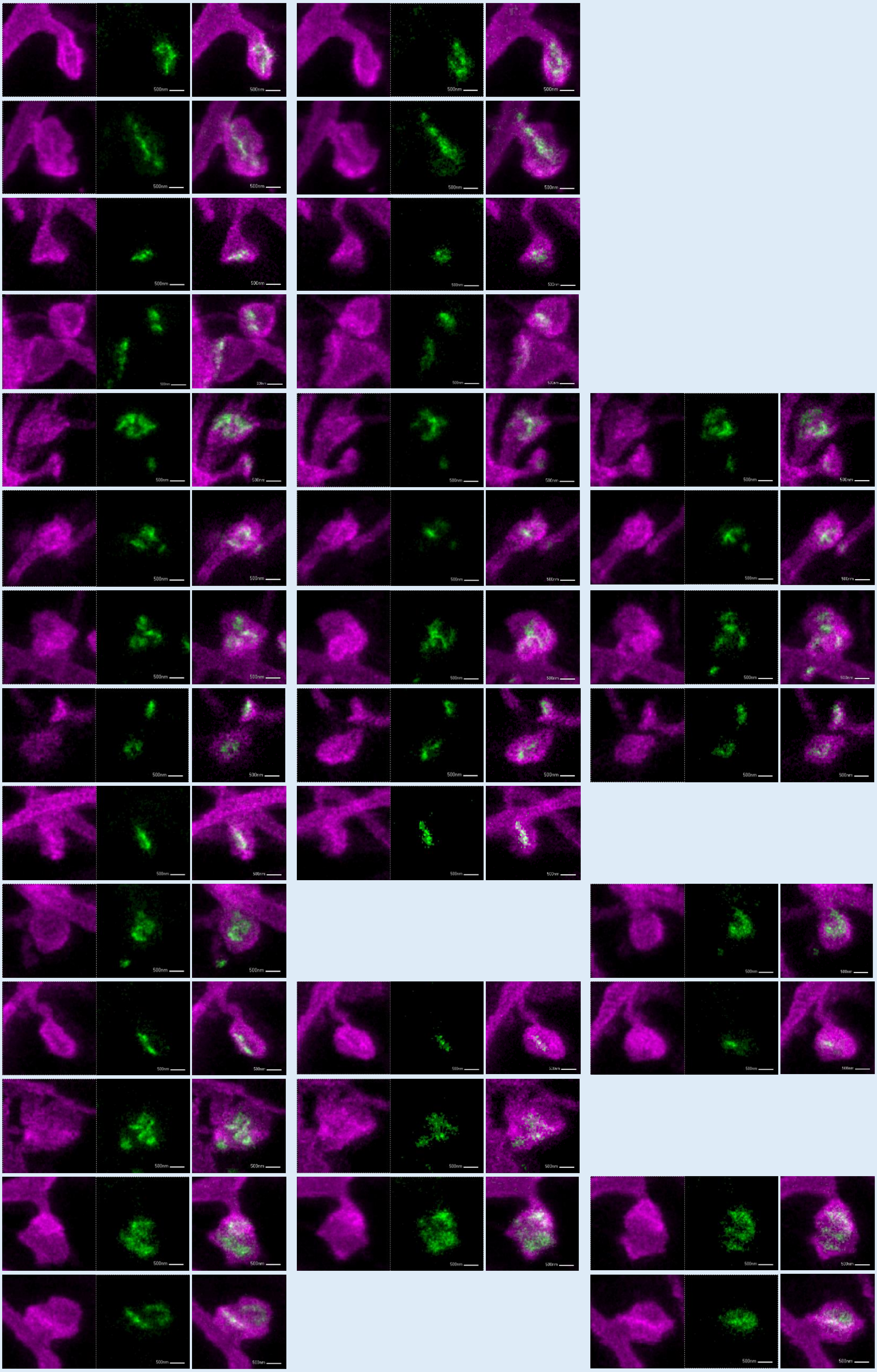

A5 - EE

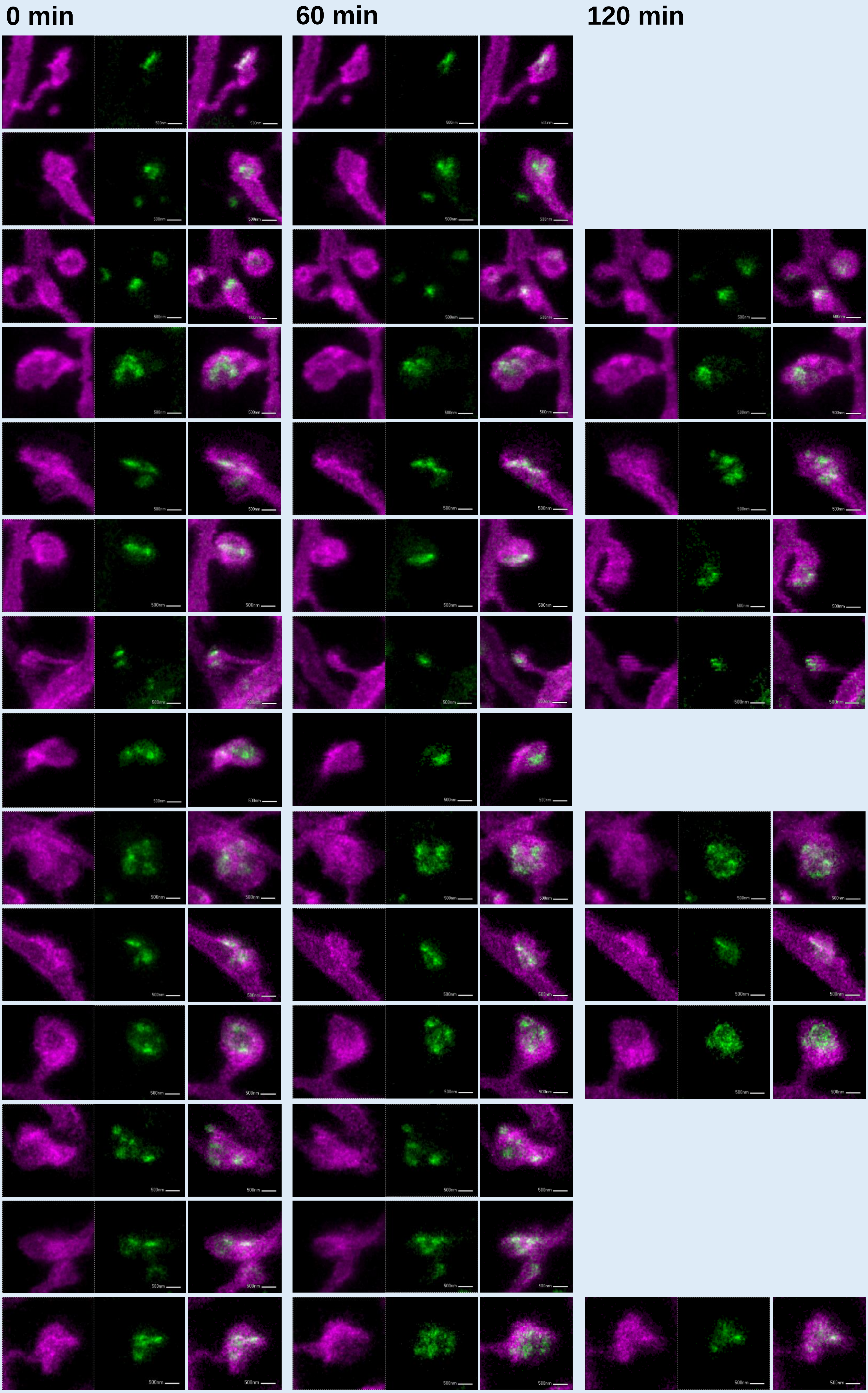

A6 - EE

0 min **60 min** **120 min**

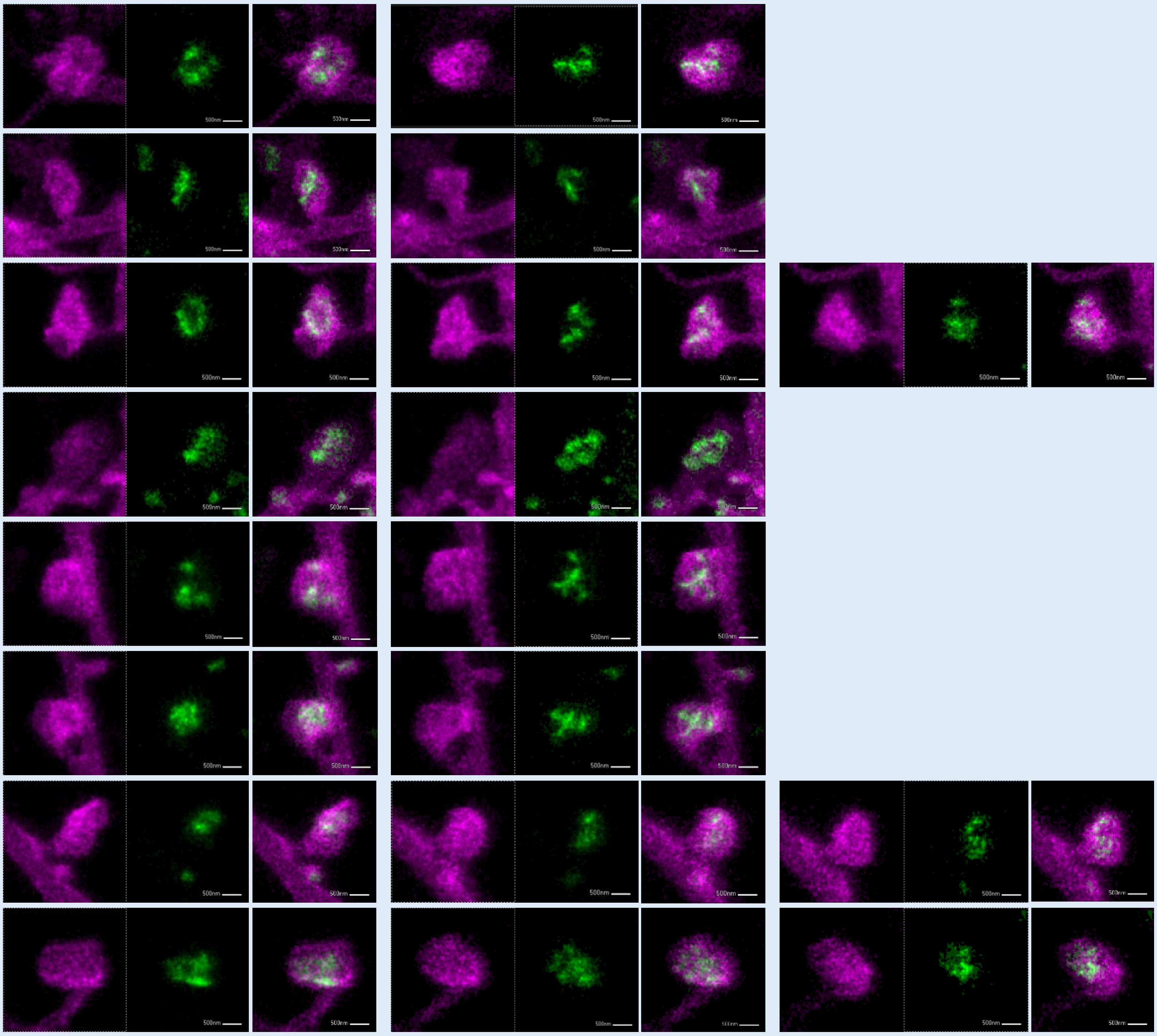

A7 - EE

0 min **120 min**

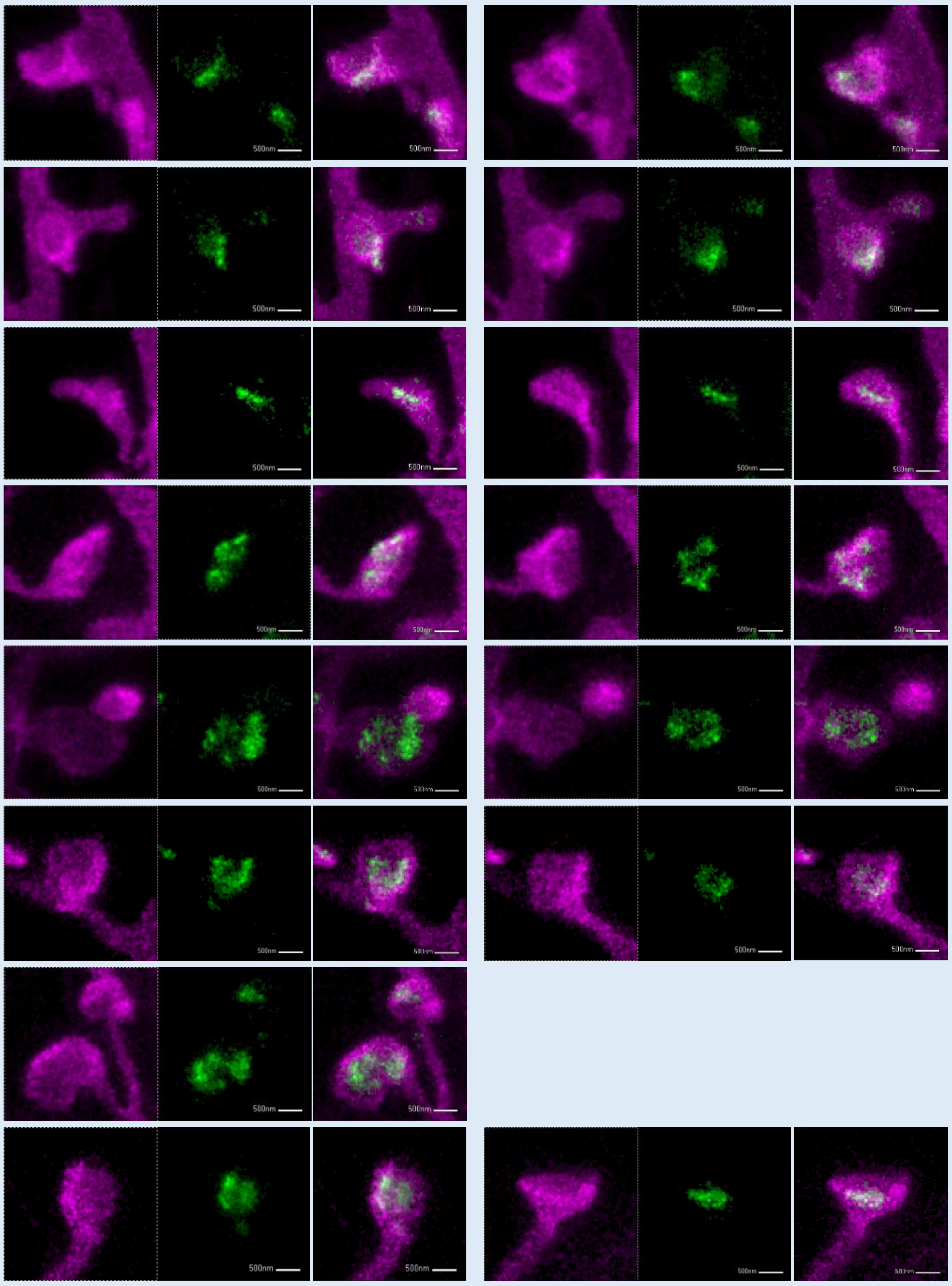

B1 - Ctr

0 min **30 min** **60 min**

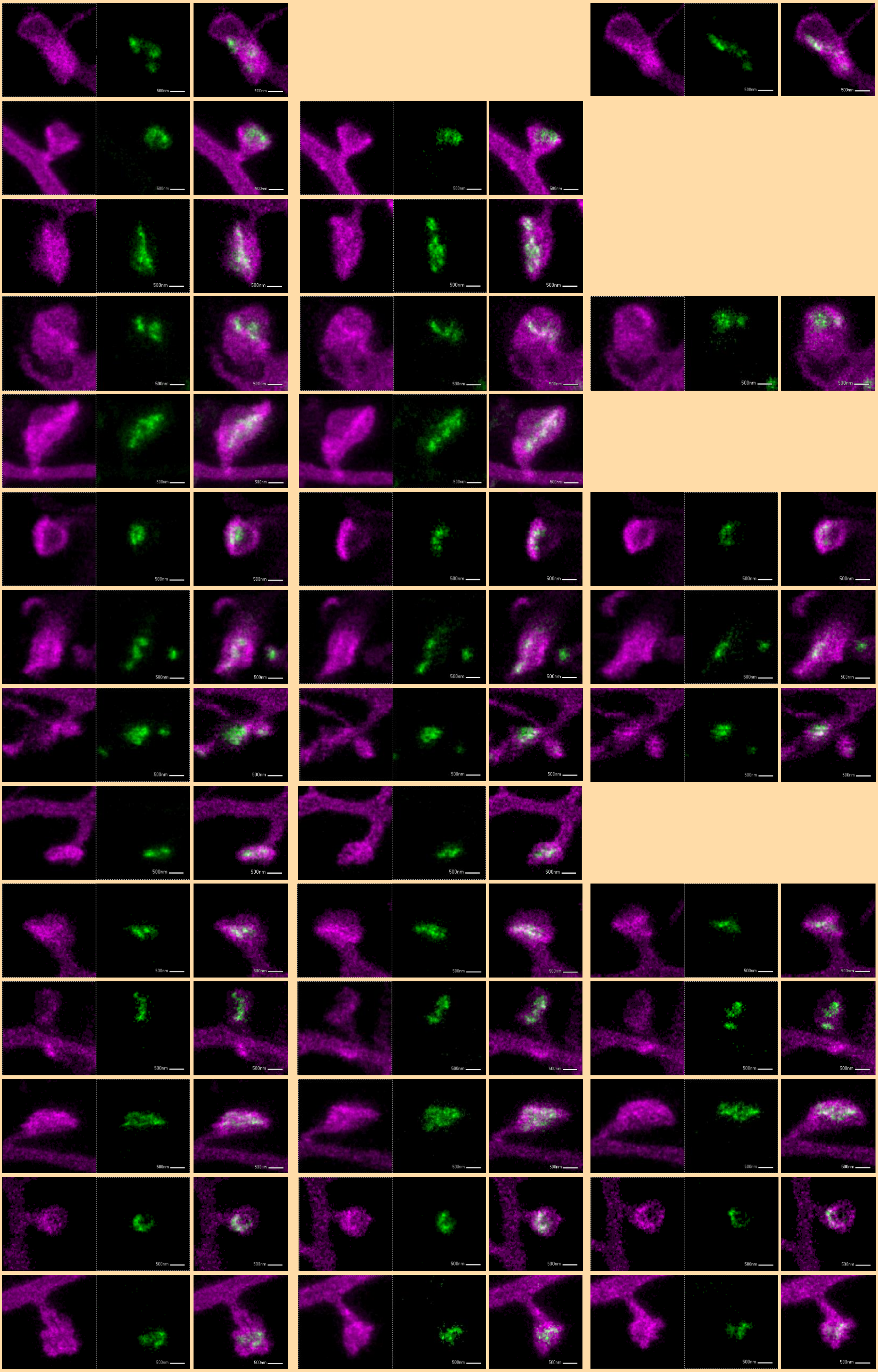

B2 - Ctr

0 min 30 min 60 min

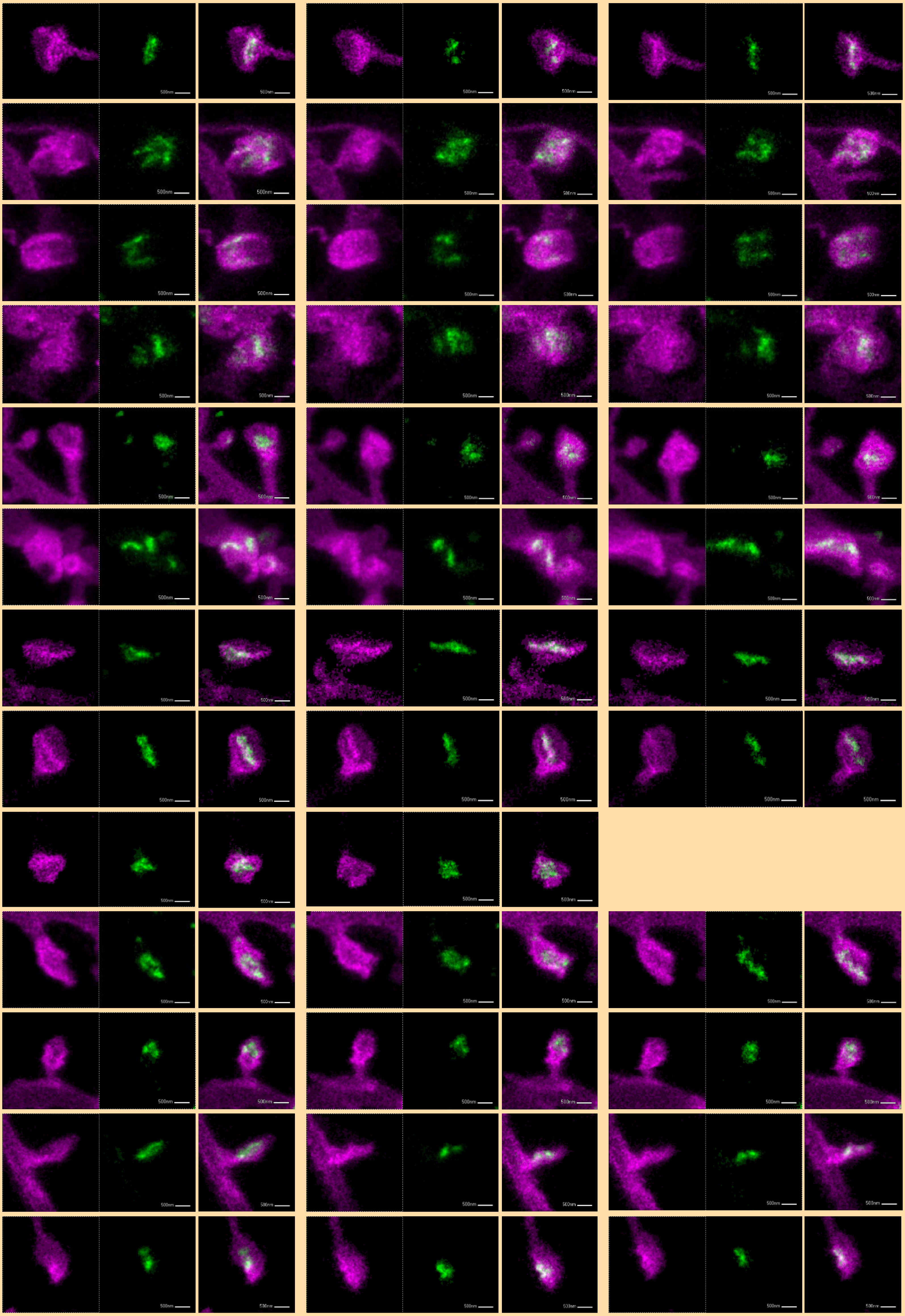

B3 - Ctr

0 min **60 min** **120 min**

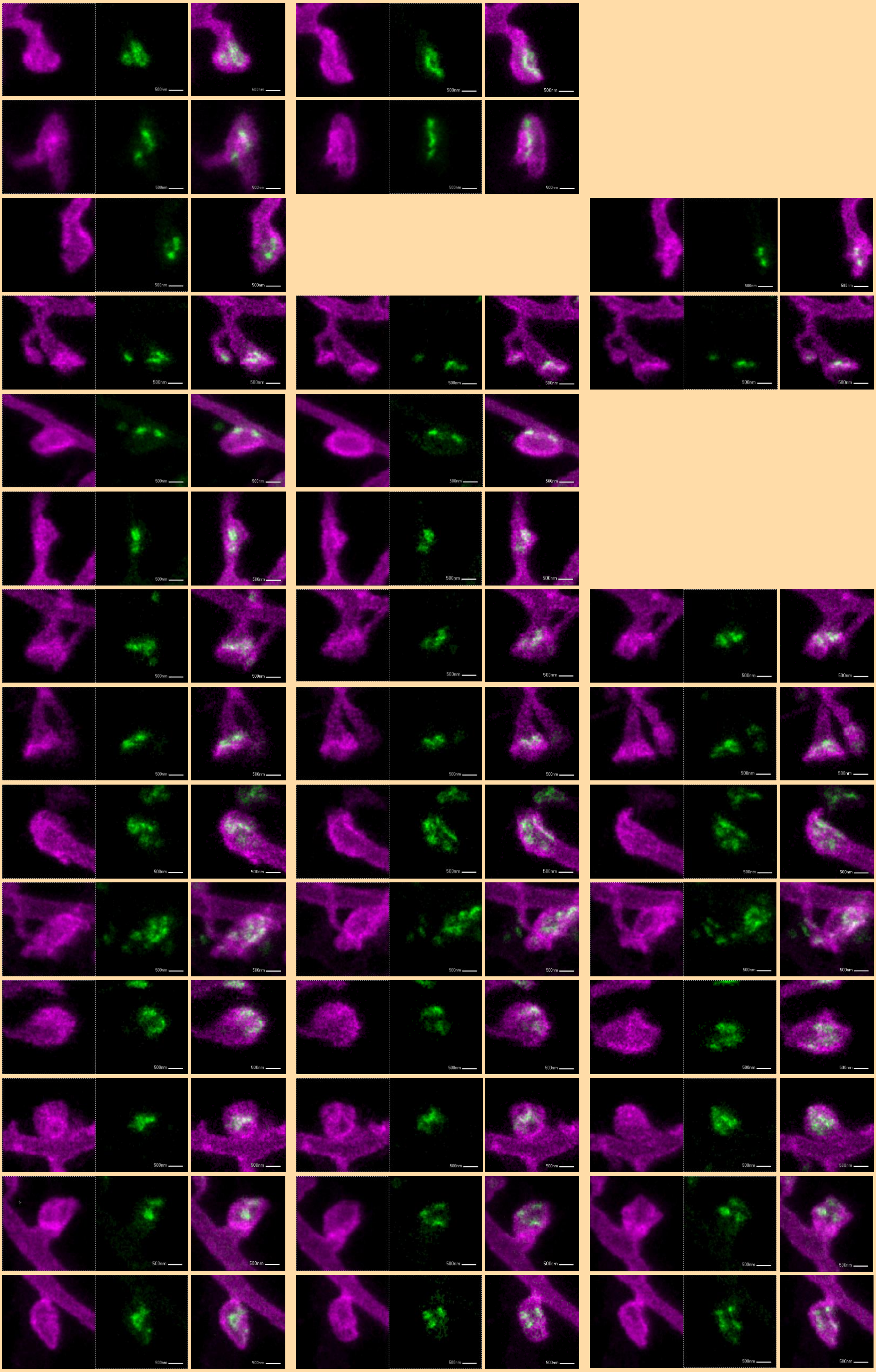

B4 - Ctr

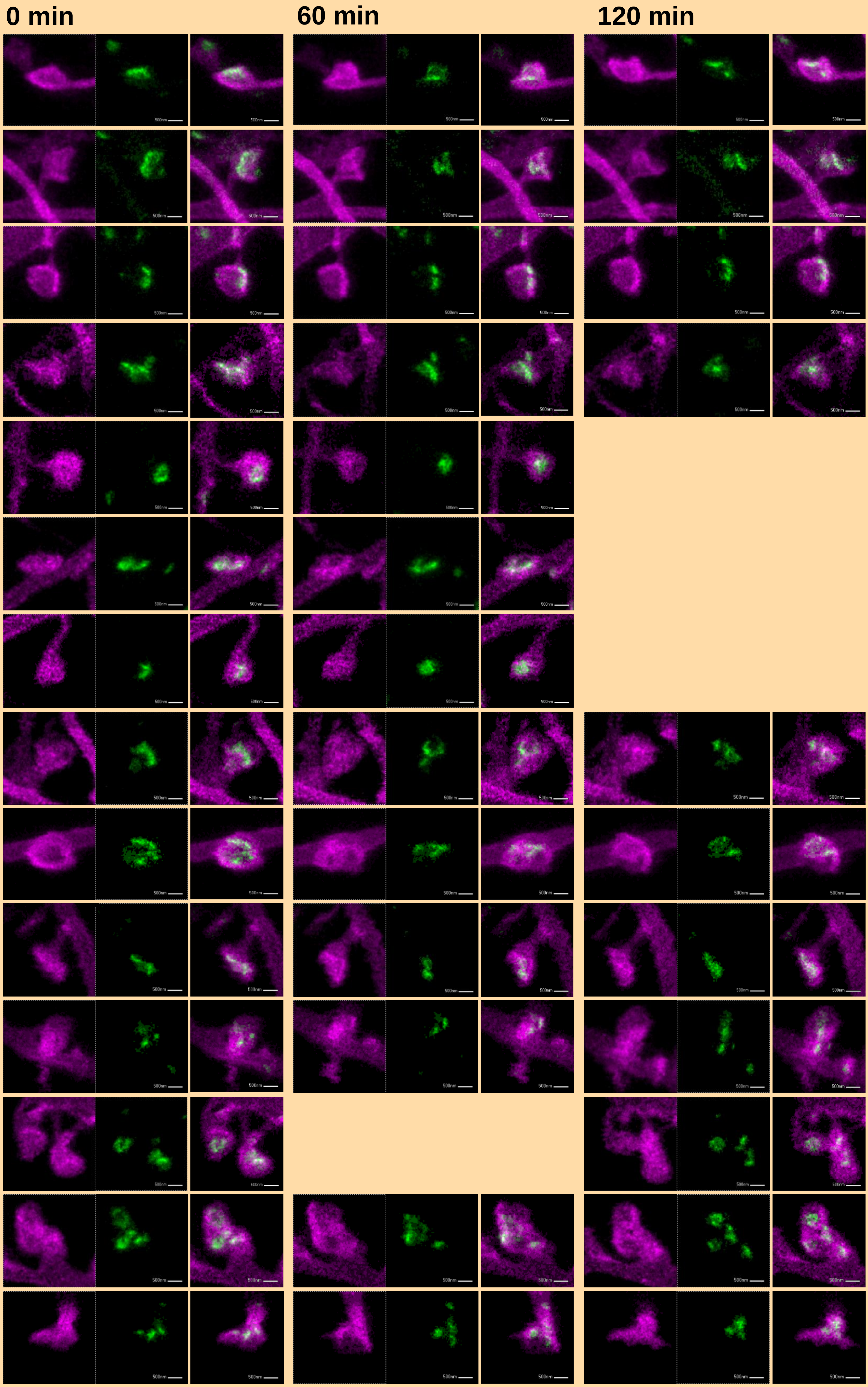

B5 - Ctr

0 min **60 min** **120 min**

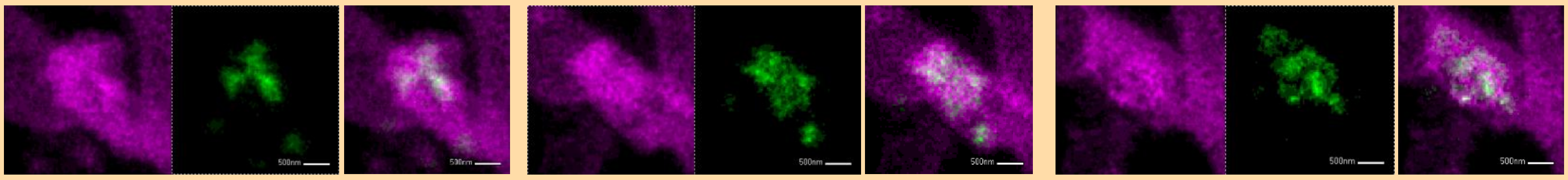

0 min **120 min**

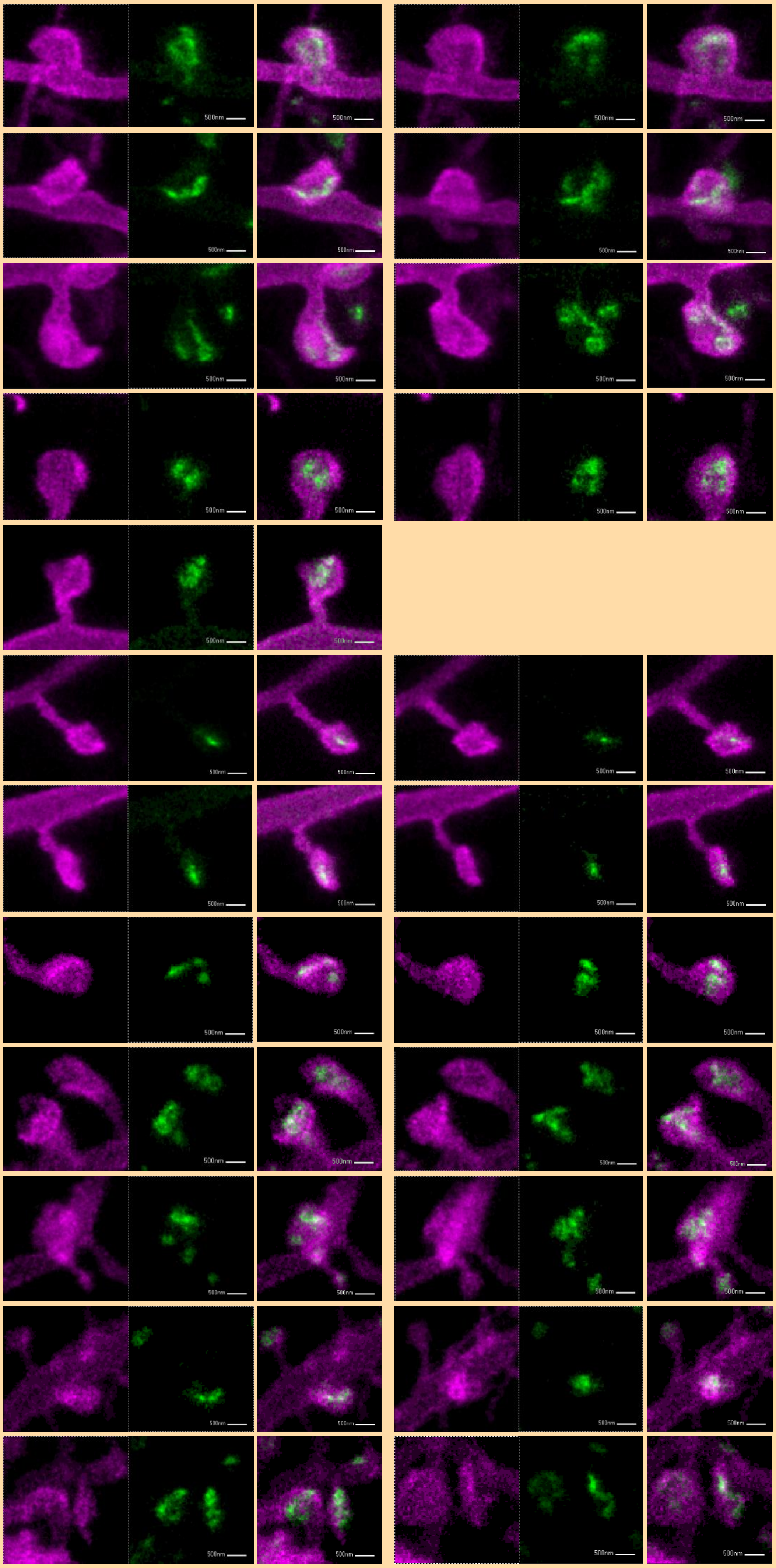

B6 - Ctr

0 min **120 min**

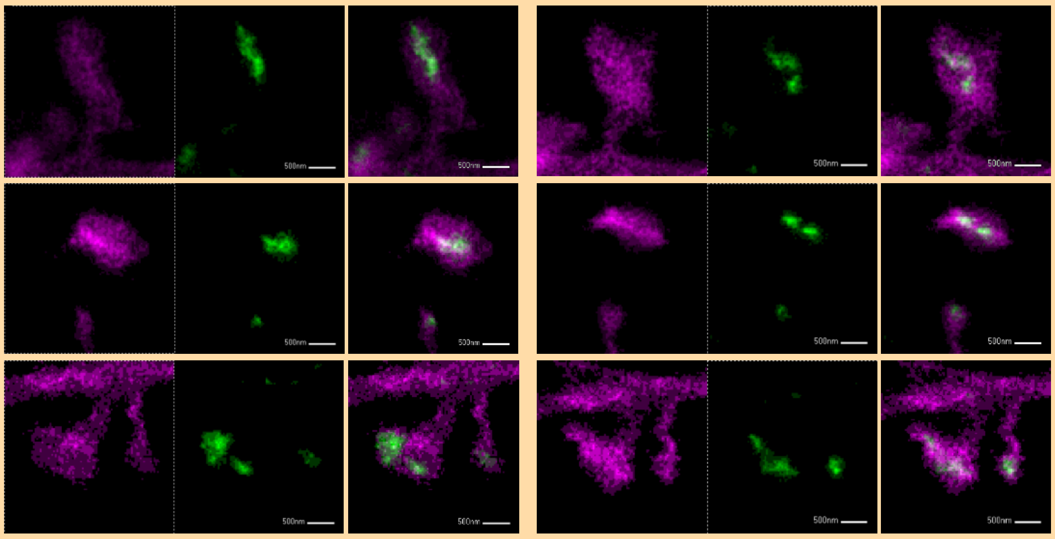

Supplement: Figure 5—source data 1. [file elife-73603-fig5-data1.pdf]
